# Keeping Pace with Your Eating: Visual Feedback Affects Eating Rate in Humans

**DOI:** 10.1371/journal.pone.0147603

**Published:** 2016-02-01

**Authors:** Laura L. Wilkinson, Danielle Ferriday, Matthew L. Bosworth, Nicolas Godinot, Nathalie Martin, Peter J. Rogers, Jeffrey M. Brunstrom

**Affiliations:** 1 Nutrition and Behaviour Unit, School of Experimental Psychology, University of Bristol, Bristol, United Kingdom; 2 Behavior and Perception group, Nestlé Research Centre, Lausanne, Switzerland; University G. d'Annunzio, ITALY

## Abstract

Deliberately eating at a slower pace promotes satiation and eating quickly has been associated with a higher body mass index. Therefore, understanding factors that affect eating rate should be given high priority. Eating rate is affected by the physical/textural properties of a food, by motivational state, and by portion size and palatability. This study explored the prospect that eating rate is also influenced by a hitherto unexplored cognitive process that uses ongoing perceptual estimates of the volume of food remaining in a container to adjust intake during a meal. A 2 (amount seen; 300ml or 500ml) x 2 (amount eaten; 300ml or 500ml) between-subjects design was employed (10 participants in each condition). In two ‘congruent’ conditions, the same amount was seen at the outset and then subsequently consumed (300ml or 500ml). To dissociate visual feedback of portion size and actual amount consumed, food was covertly added or removed from a bowl using a peristaltic pump. This created two additional ‘incongruent’ conditions, in which 300ml was seen but 500ml was eaten or vice versa. We repeated these conditions using a savoury soup and a sweet dessert. Eating rate (ml per second) was assessed during lunch. After lunch we assessed fullness over a 60-minute period. In the congruent conditions, eating rate was unaffected by the actual volume of food that was consumed (300ml or 500ml). By contrast, we observed a marked difference across the incongruent conditions. Specifically, participants who saw 300ml but actually consumed 500ml ate at a faster rate than participants who saw 500ml but actually consumed 300ml. Participants were unaware that their portion size had been manipulated. Nevertheless, when it disappeared faster or slower than anticipated they adjusted their rate of eating accordingly. This suggests that the control of eating rate involves visual feedback and is not a simple reflexive response to orosensory stimulation.

## Introduction

Deliberately eating slowly appears to promote satiation [1–3] and foods that are eaten quickly tend to be eaten in larger portions [4, 5] and have lower expected satiation [6]. For a recent systematic review see Robinson *et al*. [2]. These acute effects might also accumulate over time. People who eat at a faster rate tend to have a higher body mass index (BMI) [7–9]. Indeed, a recent clinical intervention suggests that a reduction in eating rate produces a significant and sustained (12-months post treatment) reduction in BMI and body fat in adolescents [10]. Despite its importance, rather little is known about the process that governs our rate of eating and the mechanism that supports a relationship between eating rate and meal size.

Simply infusing food directly into the stomach has very little effect on appetite and fullness [11]. Broadly, this tells us that the process of eating plays a causal role in satiation. One possibility is that eating quickly reduces the level of orosensory stimulation (sometimes referred to as orosensory exposure). In animal studies, Davis and colleagues have shown that orosensory stimulation can both inhibit and promote food intake [12]. When food is allowed to drain from the stomach via a gastric fistula then meal size increases dramatically. However, and critically, the initial post-surgery meal is not dissimilar to that observed in real-feeding animals. This demonstrates that in the absence of other feedback, orosensory stimulation can inhibit food intake. However, over time, ‘sham feeding’ animals relearn that orosensory stimulation no longer reliably predicts the ingestion of food, and in the absence of this ‘conditioned satiation,’ meal size increases [13].

Observations of this kind are very revealing. However, they also generate broader questions about ways in which orosensory stimulation and oral metering might operate in humans. Unlike other animals, we often plan our meal size in advance [14, 15]. Therefore, the need to rely on direct orosensory feedback in the control of meal size may have been largely superseded by an ability to plan and to use other pre-ingestive cues based on semantic and visual information, both before and during a meal. Accordingly, several studies show that information about a food can have a meaningful effect on feelings of fullness after it has been consumed [16–18].

In one study a passive self-refilling soup bowl was used to remove visual feedback during a meal. Remarkably, meal size increased by 73% yet had little effect on satiation [19]. Consistent with this role for ‘meal monitoring,’ distraction appears to reduce the fullness that is experienced at the end of a fixed meal [20, 21]. Importantly, we note that distraction has also been found to affect eating rate [22, 23], as has hunger [24, 25], portion size [26, 27], and palatability [28, 29]. For example, when presented with a larger portion of the same food, participants take larger bite sizes and demonstrate a faster eating rate [27]. Together, these observations support a novel theoretical proposition. As noted above, eating rate has been thought to influence satiation by moderating orosensory stimulation. In this context, researchers have tended to focus on the physical and textural properties of food (e.g., [4, 30, 31]). However, these effects of distraction, hunger, portion size, and food liking highlight an alternative (but not mutually exclusive) possibility—that eating rate is also under cognitive control. This study explores the prospect that rate of eating might also be governed by a hitherto unexplored process that uses ongoing estimates of the volume of food remaining in a container to adjust food intake during a meal. Consistent with this idea, it appears that manipulations to perceived volume have a marked effect on the rate of beer consumption [32]. In the present study, we sought to determine whether a similar process operates when eating a semi-solid food.

To explore this idea, we manipulated visual information about the amount of food consumed in a meal. This was achieved using a peristatic pump that controlled the amount of food that covertly entered or left a bowl as participants ate. In congruent conditions, the participants saw and consumed either 300ml or 500ml. In incongruent conditions, the participants saw either 300ml or 500ml at the start of the meal and then went on to consume a different amount, either 500ml or 300ml. At a constant eating rate, the volume in the bowl will reduce faster in the see 500ml/eat 300ml condition than in see 300ml/eat 500ml condition. If eating rate is governed by a process that meters intake and corrects for faster- or slower-than-anticipated changes in perceived volume then we would expect to observe compensation for the mismatch. Accordingly, we anticipated a faster eating rate in the see 300ml/eat 500ml condition relative to the see 500ml/eat 300ml condition, and for eating rate to be at an intermediate pace in the two congruent conditions. To demonstrate that this effect generalises across different types of semi-solid food, we compared eating rate using both a savoury soup (tomato soup) and a sweet dessert (custard).

## Materials and Methods

### Experiment overview

We decided to test 80 participants in a between-subjects design. On arrival they were shown either 300ml or 500ml of tomato soup or custard. Participants then consumed either a 300ml or 500ml portion. An orthogonal combination of seeing either 300ml or 500ml and then eating either 300ml or 500ml and two different food types rendered eight separate conditions (10 participants allocated to each condition). For each food type (custard and tomato soup), there were two congruent conditions (participants both saw and ate the same amount—either 300ml or 500ml) and two incongruent conditions (participants saw and ate different amounts—they either saw 300ml and ate 500ml or they saw 500ml and ate 300ml). Incongruent eating was achieved by covertly manipulating the amount of soup or custard entering or leaving the bowl during the meal. Eating duration was recorded. In addition, measures of hunger and fullness were taken immediately before the meal and periodically for 60 minutes after it had been consumed.

### Participants

Eighty participants (50 women and 30 men) were recruited from the staff and student populations at the University of Bristol (United Kingdom). To reduce demand awareness, participants were told that the purpose of the study was to determine ‘do liquid foods quench thirst?’ Prior to participation, participants were informed that they could not take part in the study if they; i) were vegan, ii) were allergic to custard or tomato soup, or iii) had previously assisted with studies involving our self-refilling/draining soup-bowl apparatus. Our sample had a mean age of 24.8 years (*S*.*D*. = 8.7; Range = 18–62) and a mean BMI of 23.2 kg/m^2^ (*S*.*D*. = 3.8; Range = 16.3–40.4). All participants received £5 (Sterling) for their assistance. Ethical approval was granted by the University of Bristol’s Faculty of Science Human Research Ethics Committee and all participants provided written informed consent before assisting with the study.

### Test foods and apparatus

**Fig 1:**
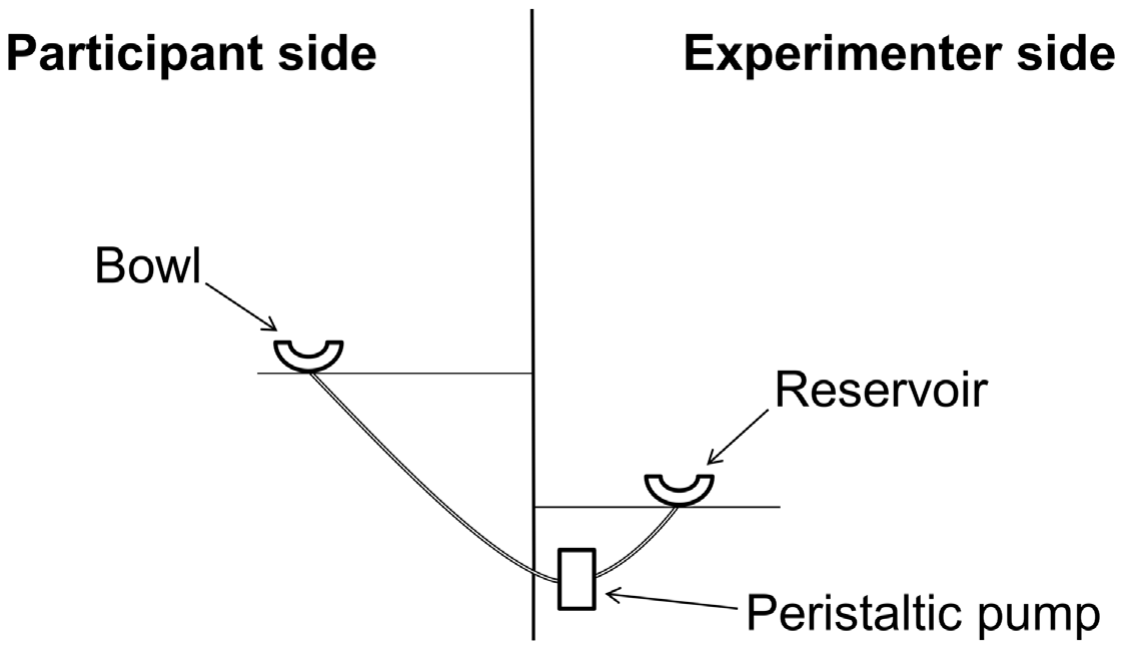
Depiction of the self-refilling soup bowl apparatus.

Following a previous study [33], soup or custard was added or removed from a transparent bowl using a peristaltic pump (see Fig 1). The bowl was presented in front of the volunteers and it was fixed to a table. A tall screen was positioned at the back of the table. This separated the participant from both the experimenter and a second table, which supported the pump and a reservoir containing the test food. Throughout the experiment, the volunteers were unable to see the pump or the reservoir.

The bottom of the transparent bowl was connected to a length of temperature-insulated food-grade tubing. The tubing fed through a hole in the table (1cm diameter; immediately under the bowl) and connected to the pump and then to a reservoir of either soup or custard via a hole in the screen. This set up was hidden from the participants using a heat-proof cover and a tablecloth. The experimenter manipulated the direction and flow rate using an adjustable motor controller that was attached to the pump. With experience, the experimenter was able to monitor eating rate and to match this with a flow rate that ensured that the pump was in operation over the duration of the meal. Both the soup and the custard were consumed using a dessert spoon.

Two different test foods were included in this experiment–one savoury and one sweet. The savoury food was a warm ‘Sainsbury’s Basics creamed tomato soup’ (39 kcal / 100 g, Sainsbury’s Supermarkets Ltd, London, U.K.). The sweet food was a warm ‘Sainsbury’s Basics instant custard’ (77 kcal / 100 g, Sainsbury’s Supermarkets Ltd, London, U.K.).

### Measures

#### Hedonic ratings

Depending on condition, a taster portion (10ml) of either tomato soup or custard was provided in a 20ml glass bowl with a teaspoon. Using a computer, participants were asked to rate the pleasantness and their desire-to-eat the sample. Specifically, participants were shown 100-mm visual-analogue rating scales and were asked ‘How PLEASANT is the taste of this food right now?’ and ‘How strong is your DESIRE TO EAT this food right now?’ with anchor points ‘Not at all’ and ‘Extremely.’ All measures were elicited using custom software (available from the authors on request) written in Visual Basic (version 6.0 distributed by Microsoft; Microsoft Corp, Redmond, WA).

#### Eating rate

Participants were instructed to start eating at their own time of choosing and to eat at their own natural pace. They were also told that they should stay in the booth when they had finished their meal. A stopwatch was placed in each sensory booth. Participants were instructed “You will see that there is a stop watch in your booth. We would like you to press start on the stop watch when you begin eating and press stop on the stop watch when you have reached the lower black line.” Therefore, they were aware that meal duration was being monitored. However, they were told that “we are asking people to do this because some people eat at different paces to others so we have to make a note so we know who is who. We need you to operate the stop watch because we don’t want to be looking over your shoulder while you are eating.”

#### Assessment of hunger, fullness and thirst

Computerised 100-mm visual-analogue rating scales were used to assess hunger (Heading: 'How hungry do you feel right now?'; Anchor points: 'Not at all hungry' and 'Extremely hungry') and fullness (Heading: 'How full do you feel right now?'; Anchor points: 'Not at all full' and 'Extremely full'). From each pair of values, a composite 'fullness score' was calculated using the formula ((100 –hunger) + fullness)/2). Ratings were taken at the beginning of each session and immediately after eating. Participants were also given further sets of hunger and fullness ratings 20 minutes, 40 minutes, and 60 minutes after meal termination. To generate a single 'satiety score,' for each participant, the total area under the curve (AUC) was calculated based on composite fullness scores at 0, 20, 40, and 60 minutes. AUC was computed using the trapezoidal rule. Thirst was also rated in combination with hunger and fullness. This measure was included only to reinforce the cover story and to check for baseline differences across groups.

### Procedure

Participants were asked to abstain from eating for three hours prior to arrival at their test session. Testing took place on weekdays between 11:00 and 14:45 hours. On arrival, the participants read an information sheet and signed a consent form. Baseline ratings of hunger, fullness, and thirst were taken. Participants were then asked to provide hedonic ratings for their allocated test food. They were then taken to a testing booth where a bowl of one of the foods was waiting. They were instructed to avoid touching the bowl and to eat until the volume of soup/custard remaining matched a line on the side of the bowl. The line ensured that eating terminated with 100ml of soup/custard remaining, thereby obscuring the bottom of the bowl. To accommodate for this amount, across conditions, the initial starting portion was 100ml greater than the amount to be consumed. All participants were informed that eating their prescribed portion was a mandatory part of the procedure. In the incongruent conditions, the peristaltic pump was switched on at the beginning of the meal. At the end of the meal the participants again rated their hunger, fullness, and thirst. After 20 minutes, these ratings were repeated and participants completed the Three Factor Eating Questionnaire (TFEQ) [34]. Further sets of ratings were issued after 40 minutes and 60 minutes. Over this period the participants were permitted to engage in light reading. At the end of the session the participants were asked to respond to the question ‘What was the aim of the experiment?’ No participants demonstrated any level of demand awareness. Finally, a measure of height and weight was taken. Participants were fully debriefed at the end of the study by email. Testing sessions lasted approximately 75 minutes.

### Data analysis

In the first instance, the raw data were converted to *z*-scores and screened for outliers. Scores falling outside 99.9% of a normal distribution were entered as missing data [35]. We found no outliers in our measure of eating duration. Only one rating was classed as an outlier. This pleasantness rating was entered as a missing datum.

To explore across-condition differences in baseline measures, hedonic ratings of the test food, and participant characteristics, we ran ten 2 (food type; custard or tomato soup) x 2 (amount eaten; 300ml or 500ml) x 2 (amount seen; congruent, incongruent) between-subjects ANOVAs. Analyses were performed on baseline fullness, baseline thirst, pleasantness of the test food, desire-to-eat the test food, BMI, age, gender, TFEQ restraint, TFEQ disinhibition, and TFEQ hunger.

For each meal, a measure of eating rate was calculated by dividing the amount eaten (ml) by the duration (s) of the meal. To explore effects on eating rate we ran a 2 (food type; custard or tomato soup) x 2 (amount eaten; 300ml or 500ml) x 2 (amount seen; congruent, incongruent) between-subjects ANOVA. Tukey’s *post hoc* analysis was used to explore the interaction between amount eaten and amount seen. Specifically, eating rate was compared across amounts eaten (300ml *vs* 500ml) in the congruent and incongruent contexts, separately. We also carried out comparisons of eating rate when the amount seen was congruent or incongruent with the amount consumed—separate tests were carried out for 300ml and 500ml portions. We also recognise an alternative approach in which measures of meal duration are assessed rather than eating rate (ml/s). Since eating rate is derived from meal duration both approaches yield near identical results. Where we observed significant findings relating to eating rate we confirmed the same result in an analysis of meal duration.

An identical data analysis strategy was used to explore the effect of amount seen and amount eaten on composite fullness scores immediately after eating and on the satiety scores derived from responses over a 60-minute post-meal duration.

All data were analysed using IBM SPSS statistics version 21 (IBM, New York, USA). In all analyses we applied a critical *p*-value of < .05.

## Results

### Baseline measures, hedonic ratings, and participant characteristics

Table 1 details baseline measures and participant characteristics (means ± *S*.*D*.) for the eight groups, separately. S1 Table (see supporting information) provides statistical values associated with the ten 2 (food type; custard or tomato soup) x 2 (amount eaten; 300ml or 500ml) x 2 (amount seen; congruent, incongruent) between-subjects ANOVAs. All *F* ratios failed to reach statistical significance with three exceptions. In each case, we suspect these can be attributed to chance. First, we observed a significant main effect of food type on age (years)–participants who ate the tomato soup were older (M = 26.7 years, *S*.*D*. = 11.0) than participants who ate the custard (M = 22.9 years, *S*.*D*. = 5.0). Second, we found a significant interaction between food type (tomato soup or custard) and amount seen (congruent or incongruent) in TFEQ-hunger scores. For custard, participants who ate in the congruent condition had higher scores than those who ate in the incongruent condition. By contrast, for tomato soup, we found the converse. Both of these baseline differences do not relate to our key hypotheses and are theoretically uninteresting. Third, for desire-to-eat (mm), we observed a significant interaction between amount eaten (300ml or 500ml) and amount seen (congruent or incongruent). In the eat 300ml condition participants showed a greater desire to eat the test food in the incongruent condition (M = 75.7 mm, *S*.*D*. = 23.0) than in the congruent condition (M = 56.7 mm, *S*.*D*. = 26.4). By contrast, in the eat 500ml condition, responses were broadly similar in the case of congruent and incongruent eating (respectively, M = 78.4 mm, *S*.*D*. = 12.4 and M = 71.1 mm, *S*.*D*. = 24.4).

**Table 1:** Mean (± *S*.*D*.) scores of baseline fullness (mm), baseline thirst (mm), pleasantness of the test food (mm), desire-to-eat the test food (mm), BMI (kg/m^2^), age (years), TFEQ restraint, TFEQ disinhibition, and TFEQ hunger, in each condition, separately. The number of males and females in each condition are also displayed.

	Congruent				Incongruent			
	*Tomato soup*		*Custard*		*Tomato soup*		*Custard*	
	See 300 Eat 300	See 500 Eat 500	See 300 Eat 300	See 500 Eat 500	See 300 Eat 500	See 500 Eat 300	See 300 Eat 500	See 500 Eat 300
Baseline fullness (mm)	22.1 (9.8)	20.4 (14.4)	25.4 (21.4)	21.3 (13.3)	29.0 (25.0)	28.8 (20.9)	27.9 (16.1)	28.9 (18.1)
Baseline thirst (mm)	63.9 (21.0)	65.2 (26.1)	71.8 (30.1)	43.2 (25.1)	64.1 (26.2)	58.3 (9.3)	60.9 (18.3)	66.3 (27.4)
Pleasantness of the test food (mm)	60.5 (17.8)	78.6 (14.2)	71.3 (18.7)	79.1 (10.3)	71.1 (22.7)	73.5 (20.5)	83.4 (18.2)	80.8 (15.0)
Desire-to-eat the test food (mm)	59.4 (24.4)	79.0 (14.2)	54.0 (29.3)	77.7 (11.2)	65.6 (29.1)	72.1 (27.1)	76.5 (18.4)	79.3 (18.7)
BMI (kg/m^2^)	23.3 (2.6)	21.9 (2.7)	22.9 (4.3)	25.3 (6.5)	23.5 (4.7)	22.4 (1.7)	23.9 (3.1)	22.5 (3.2)
Age (years)	22.5 (2.1)	25.4 (12.6)	25.6 (5.9)	22.2 (4.5)	27.7 (10.0)	31.3 (14.5)	21.9 (6.1)	21.9 (2.2)
Gender	9 F / 1 M	5 F / 5 M	6 F / 4 M	6 F / 4 M	7 F / 3 M	6 F / 4 M	6 F / 4 M	5 F / 5 M
TFEQ restraint	7.6 (4.9)	6.8 (5.3)	6.4 (5.3)	8.0 (4.8)	7.7 (4.3)	7.8 (4.9)	9.9 (5.3)	8.6 (5.5)
TFEQ disinhibition	6.9 (4.2)	5.2 (4.2)	6.3 (3.0)	9.3 (2.5)	7.5 (3.0)	6.1 (3.8)	7.1 (3.1)	6.9 (4.0)
TFEQ hunger	6.7 (3.1)	4.1 (2.6)	7.6 (4.7)	6.8 (2.8)	6.4 (3.2)	6.4 (3.7)	3.7 (2.2)	5.8 (2.6)

### Eating rate

**Fig 2:**
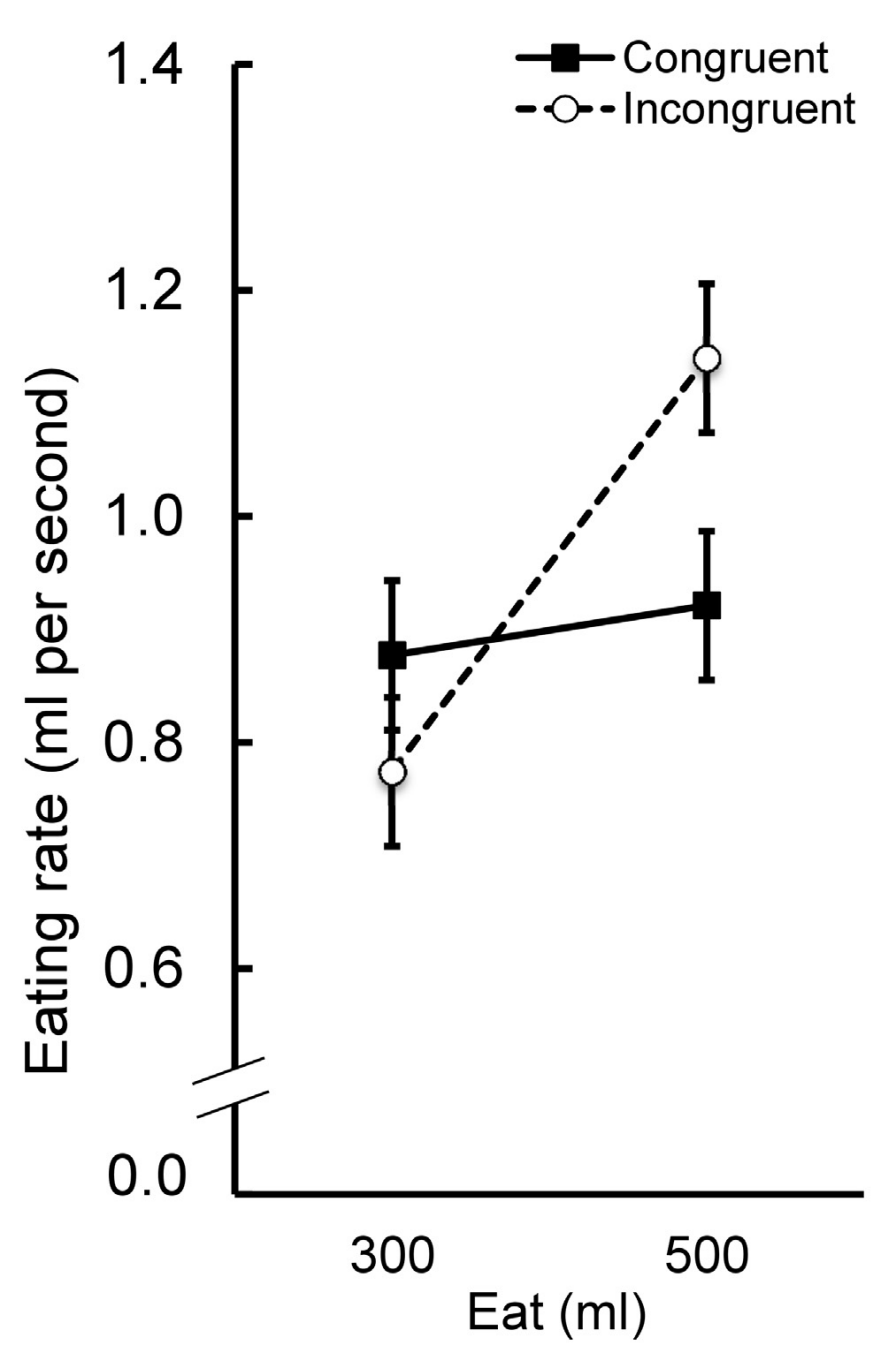
Mean (± *S*.*E*.) eating rate (ml / second) for participants in each condition.

Participants who consumed the tomato soup ate at a significantly faster rate (*M* = 1.00 ± 0.41 ml/s) than those who ate the custard (*M* = 0.85 ± 0.22 ml/s; *F*(1, 72) = 5.06, *p* = .03, *η*_*p*_^*2*^ = .07). Irrespective of food type, participants who consumed the 500ml portion ate at a significantly faster rate (*M* = 1.03 ± 0.34 ml/s) than those who received the 300ml portion (*M* = 0.83 ± 0.30 ml/s; *F*(1, 72) = 9.58, *p* = .003, *η*_*p*_^*2*^ = .12). Eating rate was not significantly different when the amount seen was congruent with the amount eaten (*M* = 0.90 ± 0.31 ml/s) or incongruent with the amount eaten (*M* = 0.96 ± 0.36 ml/s; *F*(1, 72) = 0.78, *p* = .38, *η*_*p*_^*2*^ = .01). However, the main effect of amount eaten (300ml or 500ml) was qualified by an interaction with the type of food eaten (tomato soup or custard), (*F*(1, 72) = 4.47, *p* = .04; *η*_*p*_^*2*^ = .06). Tukey's *post hoc* tests (critical difference at 5% = 0.25 and 1% = 0.30) revealed that 300ml of tomato soup was eaten at a significantly slower rate than a 500ml portion (mean difference = 0.35, *p* < .001, Cohen’s *d* = 0.93). By contrast, 300ml of custard was eaten at a similar rate as a 500ml portion (mean difference = 0.07, Cohen’s *d* = 0.29). We also found that a 500ml portion of tomato soup was eaten at a significantly faster rate than a 500ml portion of custard (mean difference = 0.29, Cohen’s *d* = 0.94). Conversely, there was no difference in eating rate between the 300ml portion of tomato soup and the 300ml portion of custard (mean difference = 0.01, Cohen’s *d* = 0.03). This interaction is theoretically uninteresting and probably reflects differences in the viscosity of the two foods. Importantly, the main effect of amount eaten (300ml or 500ml) was also qualified by an interaction with amount seen (congruent and incongruent) (See Fig 2, *F*(1, 72) = 5.92, *p* = .02, *η*_*p*_^*2*^ = .08). Note that our analysis of meal durations yielded a very similar and significant F ratio for this interaction, (*F*(1, 72) = 6.06, *p* = .02, *η*_*p*_^*2*^ = .08).

Tukey’s *post hoc* tests (critical difference at 5% = 0.25 and 1% = 0.30) revealed that participants who saw 300ml but actually consumed 500ml ate at a significantly faster rate than participants who saw 500ml but consumed 300ml (mean difference = 0.37, *p* < .001, Cohen’s *d* = 1.19). By contrast, eating rate for the 300ml and 500ml portions was not different when the amount consumed was congruent with the amount seen (mean difference = 0.04, Cohen’s *d* = 0.14). Participants who saw 300ml but consumed 500ml ate at a faster rate than those who saw 500ml and consumed 500ml. However, this effect failed to reach statistical significance (mean difference = 0.22, Cohen’s *d* = 0.68). Similarly, participants who saw 500ml but consumed 300ml ate at a slower rate than those who saw 300ml and ate 300ml but again this did not reach the criteria for significance (mean difference = 0.10, Cohen’s *d* = 0.34). Both the interaction between amount seen (congruent or incongruent) and type of food eaten (tomato soup or custard) (*F*(1, 72) = 0.47, *p* = .50, *η*_*p*_^*2*^ = .01) and the three-way interaction between type of food eaten, amount eaten and amount seen (*F*(1, 72) = 1.81, *p* = .18, *η*_*p*_^*2*^ = .03), failed to reach significance.

### Fullness

**Fig 3:**
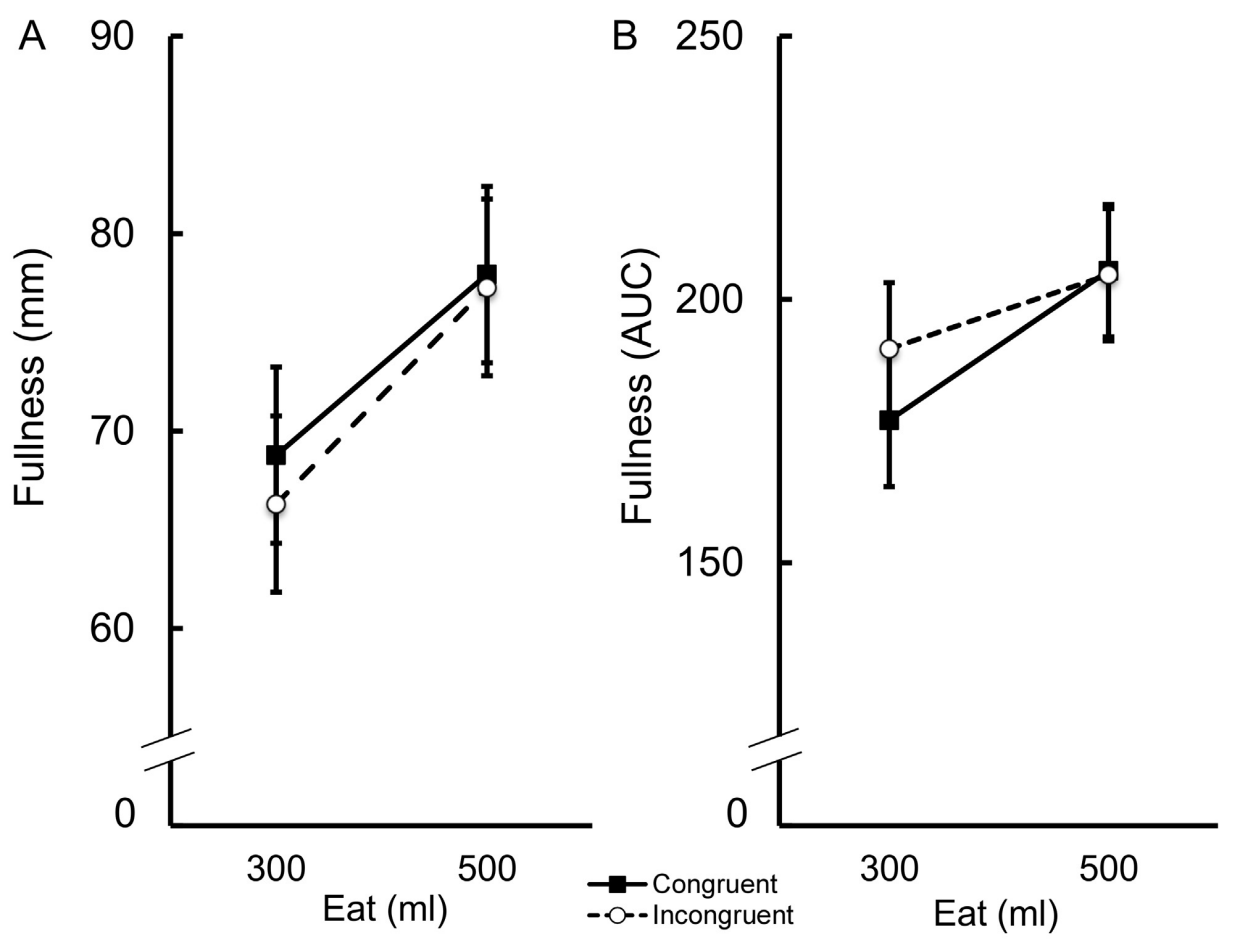
Mean (± *S*.*E*.) fullness composite scores immediately after eating (Panel A) and for an hour after eating (AUC; Panel B) the test foods. Separate values are provided for participants in each condition.

Consumption of custard was associated with greater fullness than consumption of soup, both immediately after eating (*F*(1, 72) = 7.60, *p* = .007, *η*_*p*_^*2*^ = .10) and for up to one hour after meal termination (*F*(1, 72) = 6.49, *p* = .01, *η*_*p*_^*2*^ = .08). Fig 3 shows the means and associated standard errors for fullness composite scores immediately after eating (Panel A) and satiety scores (AUC; Panel B) in each condition, separately. Irrespective of food type, participants reported greater fullness at the end of the meal if they had consumed the 500ml portion compared to participants who had eaten the 300ml portion (*F*(1, 72) = 5.08, *p* = .03, *η*_*p*_^*2*^ = .07). A similar trend was observed in our measure of satiety; participants reported greater satiety up to 60 minutes after eating the 500ml portion (*F*(1, 72) = 2.86, *p* = .10, *η*_*p*_^*2*^ = .04). There was no effect of the congruency of the amount seen with the amount eaten on fullness scores immediately after eating (*F*(1, 72) = .12, *p* = .73, *η*_*p*_^*2*^ = .002) or fullness AUC (*F*(1, 72) = .26, *p* = .61, *η*_*p*_^*2*^ = .004). In addition, the effect of congruence on fullness would be revealed by an interaction between amount seen (congruent or incongruent) with amount of food eaten (300ml or 500ml). However, this interaction was not significant, either immediately after eating (*F*(1, 72) = .04, *p* = .84, *η*_*p*_^*2*^ = .001) or for up to 60 minutes after eating (*F*(1, 72) = .33, *p* = .57, *η*_*p*_^*2*^ = .005). The interaction between amount seen and food eaten was also not significant immediately after eating (*F*(1, 72) = 2.40, *p* = .13, *η*_*p*_^*2*^ = .03) or 60 minutes later (*F*(1, 72) = 2.55, *p* = .12, *η*_*p*_^*2*^ = .03). There was also no significant interaction between amount eaten and food eaten immediately after eating (*F*(1, 72) = .33, *p* = .57, *η*_*p*_^*2*^ = .005) or 60 minutes later (*F*(1, 72) = 0.20, *p* = .66, *η*_*p*_^*2*^ = .003). Finally, the three-way interaction between amount seen, amount eaten and food eaten was not significant either immediately after eating (*F*(1, 72) = 2.91, *p* = .09, *η*_*p*_^*2*^ = .04) or 60 minutes after eating (*F*(1, 72) = 1.42, *p* = .24, *η*_*p*_^*2*^ = .02).

## Discussion

Previously, eating rate has been thought to be governed by the physical properties of a food [4, 30, 31], and by changes in hunger [24, 25] and food palatability [28, 29]. Our data suggest that eating rate (averaged across the meal) is also affected by within-meal monitoring of the amount of food consumed based on visual feedback. Specifically, we found that eating rate was unaffected by the volume (300ml or 500ml) of food consumed in the two congruent conditions. However, when we introduced a mismatch between meal size and perceived volume (the two incongruent conditions) then participants moderated their eating rate. When offered a small portion in apparently larger volume participants ate at a slower rate than they did when offered an apparently small portion in a larger volume. It is as if either consciously or unconsciously, our participants observed the portion disappear faster or slower than ideal and tempered their rate of eating accordingly. Under normal circumstances (*i*.*e*., in our congruent conditions) we may be unaware of this proactive metering of eating rate. It is only by ‘tricking’ the system (in this case, using a refilling/draining soup bowl) that this cognitive process is exposed. In the present study, we assessed average eating rate across the meal by dividing quantity consumed (ml) by meal duration (s). While this methodology is commonly employed to assess the relationship between eating rate and energy intake [2], we recognize that, in future it might be instructive to include continuous assessments of eating rate and other oral-processing behaviours throughout a meal. If the effect that we have observed is driven by participants implicitly or explicitly noticing that the bowl is emptying quicker or slower than ideal then we would predict that the difference in eating rate should become evident as the meal progresses rather than at the beginning of a meal.

The prospect that visual feedback plays this role in moderating eating rate has not been observed or considered previously. In a previous study in our lab [33], participants were asked to consume tomato soup through a self- refilling/draining soup bowl apparatus. A 2 (amount seen; 300ml or 500ml) x 2 (amount eaten; 300ml or 500ml) between-subjects design was employed (25 participants in each condition). As a control measure, meal duration was recorded using an identical methodology to the present study (self-timed by the participant). To evaluate whether our effects are robust, we decided to conduct a *post-hoc* re-analysis of this existing dataset. We observed a consistent pattern of results. Specifically, participants ate at a faster rate when confronted with the larger portion, *F*(1, 94) = 22.79, *p* < .001, *η*_*p*_^*2*^ = .20. On average, participants ate faster in the incongruent conditions, *F*(1, 94) = 4.86, *p* = .03, *η*_*p*_^*2*^ *=* .05. However, the main effect of amount eaten (300ml versus 500ml) was also qualified with a significant interaction with amount seen (congruent and incongruent) (*F*(1, 94) = 8.27, *p* = .005, *η*_*p*_^*2*^ = .081). Tukey–Kramer *post hoc* tests revealed that participants who saw 300ml but actually consumed 500ml ate at a significantly faster rate (M = 1.70 ml / s, *S*.*D*. = 0.60) than participants who saw 500ml but consumed 300ml (M = 0.99 ml / s, *S*.*D*. = 0.42; critical difference at 5% = 0.34 and 1% = 0.42). By contrast, eating rate for the 300ml (M = 1.05 ml / s, *S*.*D*. = 0.43) and 500ml (M = 1.23 ml / s, *S*.*D*. = 0.33) portions was not different when the amount consumed was congruent with the amount seen (critical difference at 5% = 0.34 and 1% = 0.41). Consistent with the trend observed in this study, eating rate for the 500ml portion was significantly faster in the incongruent condition (M = 1.69 ml / s, *S*.*D*. = 0.60) relative to the congruent condition (M = 1.23 ml / s, *S*.*D*. = 0.33; critical difference at 5% = 0.34 and 1% = 0.41). This ‘retrospective replication’ provides reassurance that the effect of visual feedback on eating rate is robust across different pools of participants.

Our findings are also consistent with recent evidence that glass shape affects the rate of consumption of alcoholic beverages [32]. In this study, beer was served in either a straight sided or a curved glass. A psychophysical procedure demonstrated that participants underestimated the half-way point, and this was the case to a greater extent in the curved glass than the straight-sided glass. In an intake test the participants took 60% longer to consume the beer from the straight glass. The authors attribute this effect to a perceptual bias–participants titrated their drinking based on perceptual judgments of volume. When these are distorted, then drinking rate is affected. Future research should explore the extent to which food container shape (*e*.*g*., the angle of different bowls with the same volume) can elicit a similar perceptual bias and affect eating rate for a fixed portion of food. The authors also note that the same effect of glass size on drinking rate was not observed when the protocol was repeated using a non-alcoholic beverage. In this study, social alcohol consumers were recruited and we suspect that they prized the opportunity to consume alcohol over a non-alcoholic beverage. Although this explanation is speculative, it is consistent with the observation that participants in the beer conditions demonstrated a greater subjective craving than participants in the soft drink conditions. This may be important, because it suggests that rate of ingestion is governed by visual information, but only when a food or beverage has a high valence. One possibility is that the tendency to consume a valued food slowly reflects an active process of ‘reward metering’ that serves to prolong the experience of eating in order to maximize the pleasure that is experienced throughout a meal. Eating quickly may be very pleasurable, but only over a short period. Eating too slowly achieves the converse. It is in this context that we envisage the ‘ideal’ eating rate that we allude to earlier. If correct, then we would expect to see greater effects of our manipulation in foods that have higher value, either by dint of their sensory-affective characteristics or maybe even their monetary value. An alternative to reward metering is that eating rate increased in response to a concern that the test meal would not be finished ‘on time.’ This assumes that people have prior beliefs about the appropriate duration of a specific meal and that they use this to guide their eating behaviour. For now, we are unable to distinguish between these alternative accounts.

Although not the primary focus of our study, we were surprised that the interaction between amount eaten (300ml versus 500ml) and amount seen (congruent versus incongruent) on eating rate was not mirrored in fullness ratings at the end of the meal. Satiation was similar irrespective of whether our participants ate a 500ml meal quickly (seeing 300ml) or at a ‘normal’ rate (seeing 500ml). Likewise, eating a 300ml meal slower than normal (seeing 500ml) had little effect on rated fullness. Previously, a clear association has been observed between eating rate and satiation [2]. However, these studies have tended to explore effects of eating rate by instructing participants to consume a single food faster or slower, or by modifying the texture of food [1–3]. In relation to this difference, it is perhaps worth noting that these manipulations generate a large difference in eating rate across conditions, typically around 60% (grams / minute; range: 17 to 143%) [2]. By contrast, a 38% difference was observed across the two incongruent conditions.

In addition, our decision to use semi-solid foods reflected practical limitations around the use of solid foods in combination with a peristaltic pump. A potential concern is that this choice of test food compromised our opportunity to expose a causal relationship between eating rate and satiation. Consistent with previous evidence [36], our semi-solid foods were consumed very quickly (approximately 7.5 minutes). The largest effect of incongruence was observed when the participants ate 500ml portions. In absolute terms this extended the meal by approximately 60 seconds. We suspect this was insufficient to generate a meaningful difference in satiation. In future, studies might explore ways to maximize the effects of incongruence on meal duration and reward metering, possibly by issuing a larger meal and/or by slowing eating rate by using a solid food form that requires mastication and bolus formation.

## Supporting Information

S1 Table Statistical values associated with the analyses of baseline measures and participant characteristics. (DOCX)
