# Supplementary material for: Keeping Pace with Your Eating: Visual Feedback Affects Eating Rate in Humans
Source: PLoS One. 2016 Feb 1;11(2):e0147603. doi: 10.1371/journal.pone.0147603 (PMC4734439; doi:10.1371/journal.pone.0147603)
Supplement: S1 Table — (DOCX) [file pone.0147603.s001.docx]

**S1 Table. Statistical values associated with the analyses of baseline measures and participant characteristics.**

|  | Main effect of food type | | Main effect of amount eaten | | Main effect of amount seen | | Food type x amount eaten interaction | | Food type x amount seen interaction | | Amount eaten x amount seen interaction | | Food type x amount eaten x amount seen interaction | |
| --- | --- | --- | --- | --- | --- | --- | --- | --- | --- | --- | --- | --- | --- | --- |
|  | *F* (1, 72) | *p* | *F* (1, 72) | *p* | *F* (1, 72) | *p* | *F* (1, 72) | *p* | *F* (1, 72) | *p* | *F* (1, 72) | *p* | *F* (1, 72) | *p* |
| Baseline fullness (mm) | .04 | .84 | .17 | .68 | 2.48 | .12 | .05 | .83 | .11 | .75 | .10 | .76 | .01 | .94 |
| Baseline thirst (mm) | .19 | .66 | 1.60 | .21 | .07 | .80 | 3.74 | .06 | .79 | .38 | 1.70 | .20 | .78 | .38 |
| Pleasantness of the test food (mm)^1^ | 3.83 | .05 | 2.72 | .10 | 1.49 | .23 | .11 | .74 | .27 | .60 | 2.64 | .11 | .94 | .34 |
| Desire-to-eat the test food (mm) | .32 | .57 | 2.85 | .10 | 1.35 | .25 | .15 | .70 | 1.52 | .22 | 6.83 | .01 | .00 | .98 |
| BMI (kg/m^2^) | 1.04 | .31 | .98 | .33 | .09 | .77 | 1.29 | .26 | .53 | .47 | .18 | .67 | .99 | .32 |
| Age (years) | 4.08 | .05 | .29 | .59 | .88 | .35 | .13 | .72 | 3.98 | .05 | .17 | .68 | 1.71 | .20 |
| Gender | .82 | .37 | .21 | .65 | .21 | .65 | .82 | .37 | .00 | 1.00 | 1.84 | .18 | .82 | .37 |
| TFEQ restraint | .44 | .51 | .20 | .66 | 1.32 | .25 | . 70 | .40 | .44 | .51 | .01 | .93 | .05 | .83 |
| TFEQ disinhibition | 1.54 | .22 | .85 | .36 | .00 | .98 | 1.24 | .27 | .97 | .33 | .01 | .92 | 3.53 | .06 |
| TFEQ hunger | .01 | .92 | 3.69 | .06 | 1.03 | .31 | .01 | .92 | 5.81 | .02 | .21 | .65 | 1.86 | .18 |

^1^One outlier for this variable was entered as a missing datum – degrees of freedom = 1, 71
